# Toxic Marine Puffer Fish in Thailand Seas and Tetrodotoxin They Contained

**DOI:** 10.3390/toxins3101249

**Published:** 2011-10-18

**Authors:** Monrat Chulanetra, Nitat Sookrung, Potjanee Srimanote, Nitaya Indrawattana, Jeeraphong Thanongsaksrikul, Yuwaporn Sakolvaree, Manas Chongsa-Nguan, Hisao Kurazono, Wanpen Chaicumpa

**Affiliations:** 1 Graduate Program in Immunology, Faculty of Medicine Siriraj Hospital, Mahidol University, 2 Prannok Road, Bangkok-noi, Bangkok 10700, Thailand; Email: monchu82@gmail.com; 2 Office for Research and Development, Faculty of Medicine Siriraj Hospital, Mahidol University, 2 Prannok Road, Bangkok-noi, Bangkok 10700, Thailand; Email: sinsru@mahidol.ac.th; 3 Graduate Program, Faculty of Allied Health Sciences, Thammasat University, Rangsit Campus, Pathumthani 12120, Thailand; Email: psrimanote01@yahoo.com.au; 4 Department of Microbiology and Immunology, Faculty of Tropical Medicine, Mahidol University, 420/6 Rajvithi Road , Bangkok 10400, Thailand; Email: tmniw@mahidol.ac.th (N.I.); tmmcs@mahidol.ac.th (M.C.-N.); 5 Department of Parasitology, Faculty of Medicine Siriraj Hospital, Mahidol University, 2 Prannok Road, Bangkok-noi, Bangkok 10700, Thailand; Email: gskmu@yahoo.co.uk (J.T.); jub922@yahoo.com (Y.S.); 6 Department of Applied Veterinary Medicine and Public Health, Obihiro University of Agriculture and Veterinary Medicine, Inada-cho, Obihiro, Hokkaido 080 8555, Japan; Email: hkurazon@obihiro.ac.jp

**Keywords:** tetrodotoxin (TTX), puffer fish, *Lagocephalus* spp., *Tetraodon* spp., *Arothron* spp., Thailand, Gulf of Siam, Andaman sea, liquid chromatography/tandem mass spectrometry (LC-MS/MS), mouse bioassay, mouse lethal unit(s)

## Abstract

A total of 155 puffers caught from two of Thailand’s seas, the Gulf of Siam and the Andaman seas, during April to July 2010 were included in this study. Among 125 puffers from the Gulf of Siam, 18 were *Lagocephalus lunaris* and 107 were *L. spadiceus* which were the same two species found previously in 2000-2001. Thirty puffers were collected from the Andaman seas, 28 *Tetraodon nigroviridis* and two juvenile *Arothron reticularis*; the two new species totally replaced the nine species found previously in 1992-1993. Conventional mouse bioassay was used to determine the toxicity in all fish tissue extracts, *i.e*., liver, reproductive tissue, digestive tissue and muscle. One of each of the species *L. lunaris* and *L. spadiceus* (5.56 and 0.93%, respectively) were toxic. All 28 *T. nigroviridis* and 2 *A. reticularis* (100%) from the Andaman seas were toxic. The toxicity scores in *T. nigroviridis* tissues were much higher than in the respective tissues of the other three fish species. Liquid chromatography/tandem mass spectrometry (LC-MS/MS) revealed that the main toxic principle was tetrodotoxin (TTX). This study is the first to report TTX in *L. spadiceus*. Our findings raised a concern for people, not only Thais but also inhabitants of other countries situated on the Andaman coast; consuming puffers of the Andaman seas is risky due to potential TTX intoxication.

## 1. Introduction

Tetrodotoxin (TTX) blocks sodium ion channel of the nerve cell membrane resulting in paresthesias, ataxia, diarrhea, vomiting, respiratory insufficiency, paralysis, and even rapid death in seriously intoxicated humans [1]. The toxicity occurs after ingesting food containing the TTX and/or toxic derivatives, usually puffer fish and occasionally other seafood such as shellfish and horseshoe crabs [2]. The poisoning is particularly common in Japan but cases are also found in many other Asian countries such as China, Taiwan, Hong Kong, Philippines, Bangladesh, and Thailand, as well as in other continents [2]. TTX (~319 Da) and its derivatives have a heterocyclic guanidine in structure. They are relatively stable to the cooking temperature [2,3,4,5]. TTX is produced by many species of marine bacteria including *Pseudomonas* sp., *Vibrio alginolyticus*, *Alteromonas tetraodonis*, *Shewanella alga*, *S. putrefaciens*, *Microbacterium arabinogalactanolyticum* and *Serratia marcescens* [6,7,8,9,10,11,12,13]. It is thought that the TTX is bio-concentrated via the food chain, symbiosis and/or parasitism in many marine organisms including several puffer species, many invertebrates (e.g., blue-ringed octopus, starfish, xanthid crab, gastropods), as well as some vertebrates (e.g., atelopid frogs, gobies, newts) [2,14,15,16]. In most marine puffers, high concentrations of TTX are found in livers and ovaries/eggs, especially during spawning, but significant amounts are also detected in digestive tissue, muscles and skin [2,14,15,16]. TTX was also found in brackish water and freshwater puffers for which the highest concentration of TTX was contained in skin [14,15,16,17,18]. The origin of TTX accumulated in the brackish water and freshwater puffers is, as yet, to be determined. 

Human cases of TTX poisoning, from eating the eggs of the horseshoe crab, *Carcinoscorpus rotudicauda*, have been documented in Thailand since 1966 [19,20]. Since then, sporadic cases and focal epidemics of this paralytic food poisoning have been reported [18,21,22,23,24,25,26,27,28]. The toxic food involved in subsequent cases of TTX poisoning was not only the eggs of the horseshoe crab, but also and more frequently, brackish water and marine puffers. A significant numbers of cases of intoxication were fatal [18,29,30,31]. Thus, selling puffers in Thailand has been forbidden since 2002. Nevertheless, the fish are still landed illegally and both the intact puffers as well as their cutlets are sold in fresh markets by unscrupulous vendors. Puffer meat, which is relatively cheap, is mixed with fish flesh for making massive amounts of marketed fish balls. Moreover, some open space grill restaurants called “Moo-Kata” which have recently become popular in Thailand sell the puffer flesh, which tastes like chicken meat but cheaper, to their buffet customers. 

Three brackish water puffer species, *i.e.*, *Tetraodon leiurus*, *T. fangi* (*T. cochinchinensis*), *T. palembangenesis*, and five marine puffers caught from Andaman seas (western Thai sea) during 1992-1993, namely *Arothron immaculates*, *Chelonodon patoca*, *Lagocephalus lunaris*, *L. sceleratus* and *Xenopterus naritus* have been implicated as the cause of the TTX poisoning among the Thais [24,30,31,32]. From August 2000 to August 2001, toxicity of two puffer species, *L. lunaris* and *L. spadiceus*, caught from Gulf of Siam and landed at the fish port in Samutsakorn province (located along the east coast, about 50 km southwest of Bangkok) were studied monthly [33]. It was found that only the *L. lunaris* was toxic while the *L. spadiceus* was not. Toxicity of the *L. lunaris* was markedly seasonal; they were toxic for nine months during March to November; August was the month that the fish had the highest TTX amounts in their tissues [33]. In this study we re-investigated the puffer species caught from the Andaman seas and the Gulf of Siam and also characterized the TTX they contained.

## 2. Materials and Methods

### 2.1. Puffer Fish

A total of 155 marine puffers (accumulated number of fish caught several different times) were included in this study. They were purchased directly from the fishermen at many fish landing ports located in different provinces of Thailand. Among them, 125 were caught from the Gulf of Siam and were landed in three provinces along the Thailand east coast: Chonburi (21 fish), Rayong (55 fish) and Samutsakorn (49 fish). The other 30 puffers were caught from the Andaman Sea and were landed in Satun province located on the west coast in the southern part of the country. Commercial fishing in the Gulf of Siam and Andaman Sea was performed by using pair trawl and push nets. The fishing area usually involves at least 12 nautical miles from the coast. The fish were transported in ice to a laboratory in Bangkok where their species were identified. Each fish was weighed before dissecting for reproductive tissue, digestive tissue, liver, muscle and skin.

### 2.2. Determination of Toxicity

Toxins were extracted from each fish tissue sample as described previously [34]. Briefly, each sample was weighed, cut into small pieces and homogenized with an ultraturrax mixer (IKA Labortachnik, Staufen, Germany). Acetic acid (0.1% w/v) was added to each preparation and each sample was boiled for 5 min and centrifuged at 11,000× *g*, 25 °C for 10 min. The clear supernatant was collected and tested for toxicity by the standard mouse bioassay [35]. Toxicity of each sample was expressed as mouse lethal units (MU) [35]. One MU is the amount of the toxin that causes death of an 18-20 gram male ICR mouse (purchased from the National Laboratory Animal Center, Mahidol University, Salaya Campus, Nakhon Pathom, Thailand) in 30 min after intraperitoneal administration [36]. This research was approved by the Siriraj Animal Care and Use Committee (SI-ACUC), Faculty of Medicine Siriraj Hospital, Mahidol University, Bangkok, Thailand (approval no. 009/2553).

### 2.3. Preparation of Test Solutions

Stock standard TTX (1 mg/mL) (Sigma-Aldrich, MO, USA) was dissolved in 10 mL of 0.1% acetic acid. Each puffer fish toxin extract, diluted 1:10 in 0.03% acetic acid, was added with ethyl acetate (1:1 v/v), mixed thoroughly and kept at 25 °C until two layers were formed. The top ether layer containing fat was discarded and the lower toxic aqueous layer was added with equal volume of 50% activated charcoal in distilled water (DW). The preparation was kept at 25 °C overnight and then centrifuged at 10,000× *g* for 10 min. The charcoal was washed with DW and the adsorbed TTX was extracted out with a solution of 1:20:79 (v/v/v) of acetic acid:methanol:DW. After centrifugation to remove the charcoal, the clear toxic supernatant was filtered through a 0.45 μm membrane (Millipore, Milford, MA, USA) before subjecting to liquid chromatography/tandem mass spectrometry (LC-MS/MS), according to the method described previously [34].

### 2.4. Liquid Chromatography/Tandem Mass Spectrometry (LC-MS/MS)

The LC was performed by using Agilent 1100 series (Palo Alto, CA, USA). The 150 × 2.1 mm ZIC-HILIC (Merck KGaA, Damstadt, Germany) column and a 20 × 2.1 mm guard column (SeQuant, Haltern, Germany) were used. The gradient program set previously [34] was followed throughout using 10 mM ammonium formate and 10 mM formic acid in ultrapure water as eluent A and 80% acetronitrile in ultrapure water containing 5 mM ammonium formate and 2 mM formic acid as eluent B. The flow rate was set at 0.25 mL per min. Esquire HCT triple-quadrupole mass spectrometer ESI source (Bruker Daltonics, Germany) was used for the MS/MS. For CID fragmentation, the *m*/*z* 320, 302 and/or 304 were selected and the amplitudes were 0.65, 0.90 and 0.90, respectively. 

## 3. Results

### 3.1. Species of Toxic and Non-Toxic Puffers and Toxicity of Tissues of the Toxic Fish

**Figure 1:**
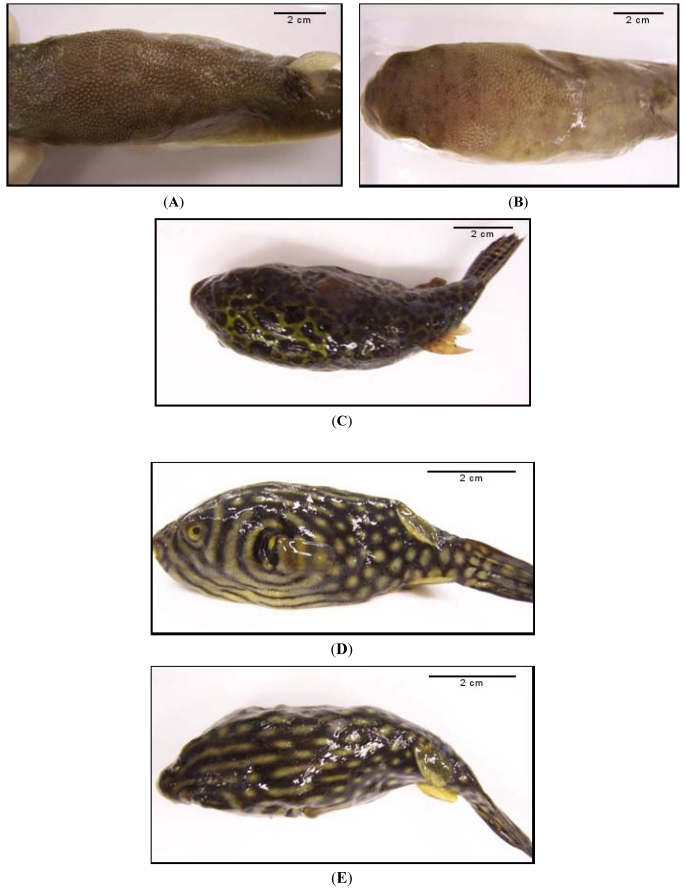
Appearances of the four puffer species caught from the Thailand seas. (**A**) Dorsal view of *L. lunaris* showing dorsal knobs which extend from head to caudal fin; (**B**) dorsal view of *L. spadiceus* which the dorsal knobs were found only on the anterior half of the back; (**C**) *T. nigroviridis* showing typical greenish skin with various sized brown spots of irregular shape; (**D**) and (**E**) side and dorsal views of *A. reticularis* showing the reticulated and striped patterns, respectively.

Toxicity of tissue extracts including liver, reproductive tissue, digestive tissue and meat of a total of 155 fish, 125 from the Gulf of Siam and 30 from the Andaman seas, were determined by the mouse bioassay. Toxicity of the fish skin extracts was not determined because the preparations became gelatin-like after being boiled in the acetic acid and could not be injected into the mouse peritoneal cavity. The 125 puffers caught from the Gulf of Siam and landed in Chonburi, Rayong and Samutsakorn provinces, were found to belong to two species, *i.e*., *L. lunaris* (18) and *L. spadiceus* (107) (Table 1). Tissue extract of only one fish each of *L. lunaris* (5.56%) and *L. spadiceus* (0.93%) contained toxin that killed the intraperitoneally injected mice. Of the 30 Andaman puffers from different fishing boats which were landed in Satun province, two species, *i.e*., *T. nigroviridis* and *A. reticularis*, were found and all of them were toxic. They were 28 fully mature *T. nigroviridis* and two small sized *A. reticularis* with poorly developed reproductive tissue suggesting that they were juvenile fish. The numbers and percentages of the toxic puffers of each species are shown in Table 1. Figure 1 illustrates the appearances of the four puffer species of this study. The *L. lunaris* had knobs on the back skin which extended from head to dorsal fin while the knobs of the *L. spadiceus* were found only at the anterior half of the back. The skin of *T. nigroviridis* is green. The *T. nigroviridis* and *A. reticularis* can be easily recognized by their typical stripes and patterns.

**Table 1:** Species of the 155 puffers caught from the Gulf of Siam and Andaman seas and number and percentage of toxic/total puffers.

Name of sea	Province	Number to toxic puffer/total number examined (%)				Total
		*Lagocephaluslunaris*	*L.spadiceus*	*Tetraodonnigroviridis*	*Arothronreticularis*	
Gulf of Siam (east coast)	Chonburi	0/2 (0)	0/19 (0)	-	-	21
	Rayong	0/11 (0)	0/44 (0)	-	-	55
	Samutsakorn	1/5 (20)	1 ^*^/44 (2.27)	-	-	49
Andaman (west coast)	Satun	-	-	28/28 (100)	2/2 ^**^ (100)	30
**Total**		**1/18 (5.6) **	**1/107 (0.93)**	**28/28 (100)**	**2/2 (100)**	**32/155 (20.6)**

^*^ Toxin was found only in the liver extract; ^*^ Both were young fish with poorly developed reproductive tissue.

Table 2 gives details of toxicity of the toxic fish tissues as determined by the mouse bioassay. All tissue extracts of the toxic *L. lunaris* contained toxin. Reproductive tissue of this fish contained highest toxicity (23.12 MU/g) followed by liver (4.16 MU/g), digestive tissue (2.46 MU/g) and muscle (2.24 MU/g). Only the liver extract of the toxic *L. spadiceus* caused death in the mice; the tissue contained 4.0 MU/g. All tissue extracts of the *T. nigroviridis* contained much higher toxicity than those of the fish from the Gulf of Siam. Only the extracts of liver, muscle and digestive tissue of the young *A. reticularis* conferred the mouse toxicity and the reproductive tissue extracts were not toxic. 

**Table 2:** Toxicity in tissues of the toxic fish determined by the mouse bioassays.

Fish species	Body weight (g)	Number of fish	Toxicity of extract (MU/g of tissue)			
			Reproductive tissue	Liver	Digestive tissue	Muscle
L. lunaris	322.79	1	23.12	4.16	2.46	2.24
L. spadiceus	108.01	1	nd	4.0	nd	nd
T. nigroviridis	88.7 ± 27.48 *	5	26.75 ± 36.82 *	51.76 ± 46.08 *	21.64 ± 21.69 *	13.74 ± 8.38 *
A. reticularis	34.96	1	nd	2.08	3.16	4.02

* mean ± SD; nd, not detectable.

### 3.2. LC-MS/MS Analysis of Puffer Fish Toxin

**Figure 2:**
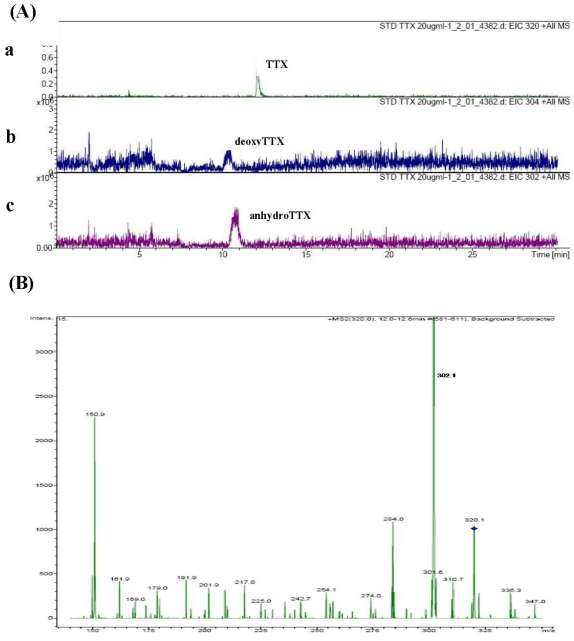
Selected ion chromatograms of standard tetrodotoxin (TTX) at *m*/*z* 320 (a), *m*/*z* 304 (b), and *m*/*z* 302 (c) (**A**); and MS/MS fragmentation pattern of TTX (*m*/*z* 320; retention time 12.0-12.6 min) (**B**).

Selected ion chromatogram of standard TTX is shown in Figure 2A. TTX (*m*/*z* 320) came out at about 12 min after sample loading (a). In addition, two other derivatives of *m*/*z* 304 and *m*/*z* 302 were detected during 10-11 min after sample loading, which are likely to be deoxyTTX and anhydroTTX, respectively (b and c). When the TTX peak (*m*/*z* 320) was subjected to MS/MS, many fragmentation products, for example, *m*/*z* 302 ([M + H − H_2_O]^+^) and *m*/*z* 284 ([M + H − 2H_2_O]^+^) were observed (Figure 2B).

**Figure 3:**
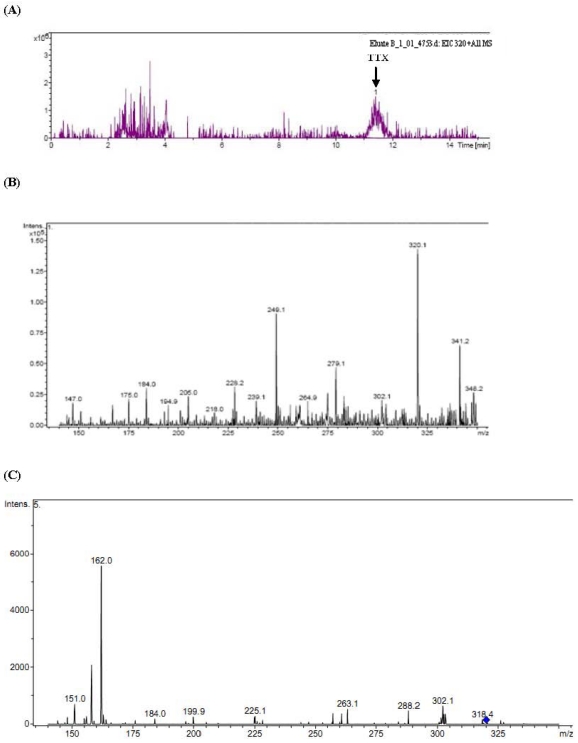
Selected ion chromatogram of the liver extract of *T. nigroviridis* at *m*/*z* 320 (**A**); MS/MS fragmentation pattern of the peak corresponding to TTX (*m*/*z* 320; retention time 11.0-11.8 min) (**B**); and MS/MS fragmentation pattern of TTX (*m*/*z* 320) in the muscle extract of *T. nigroviridis* (**C**).

Selected ion chromatogram of the liver extract of a toxic *T. nigroviridis* is shown in Figure 3A. The extract gave a peak at almost the same retention time as that of TTX. MS/MS fragmentation pattern of the peak (Figure 3B) was essentially similar to that of the standard TTX. AnhydroTTX and deoxyTTX were also detected in the extract (data not shown). Extract of muscle (the edible part) of the same *T. nigroviridis* also contained TTX, the MS/MS fragmentation pattern of which is shown in (Figure 3C).

**Figure 4:**
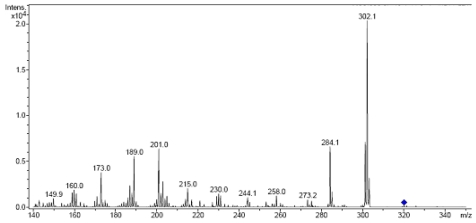
MS/MS fragmentation pattern of TTX (*m*/*z* 320) in the toxic liver extract of *L. spadiceus*.

Toxic liver extract of *L. spadiceus* was found to contain TTX and anhydroTTX (data not shown). The other tissue extracts of this fish including muscle (the edible part) and reproductive and digestive tissues did not contain detectable toxin by the mouse bioassay. Figure 4 shows the MS/MS fragmentation pattern of TTX in toxic *L. spadiceus* liver.

**Figure 5:**
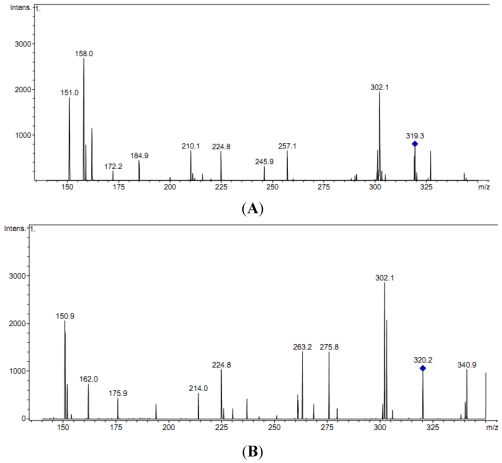
MS/MS fragmentation pattern of TTX in muscle extracts of toxic *L. lunaris* (**A**) and *A. reticularis* (**B**). The puffer tissue extracts were also analyzed for *m*/*z* 300 of saxitoxin but the toxin was not detected in any of the samples.

The fragmentation products of TTX peak (*m*/*z* 320) in muscles of *L. lunaris* and *A. reticularis* after being subjected to MS/MS are shown in (Figure 5A,B), respectively.

The puffer tissue extracts were also analyzed for *m/z* 300 of saxitoxin but the toxin was not detected in any of the samples.

## 4. Discussion

The species of 125 puffers caught several times from the Gulf of Siam in the present study were *L. lunaris* and *L. spadiceus,* which were the same species as reported previously in 2000-2001 [33]. However, liver extract of one of the previously known non-toxic *L. spadiceus* killed mice, which is the first evidence that *L. spadiceus* can be toxic. Although the mouse bioassay for detecting TTX has also been used for detecting paralytic shellfish toxin (PST) causing paralytic shellfish poisoning (PSP), the PST was not found in any of the toxic extracts of the four fish species. So, the toxicity observed in this study was from TTX. Usually, TTX has physiological function(s) in the TTX-bearing organisms and the function depends on the location of the TTX [2]. For marine puffer fish, TTX is contained in their skin and also in eggs [37,38,39]. The TTX may be the biological defense weapon to protect eggs or the fish themselves from predators [2]. The puffer eggs may also act as a source of pheromone to attract the male fish [2,40]. TTX was also found to influence ecological preference of the puffers [2]. It is believed that the puffers that contained TTX are resistant to the toxin by evolving sodium channels that are TTX-resistant, *i.e*., aromatic amino acid in the p-loop region of domain I in the TTX sensitive ion channel is replaced by a non-aromatic amino acid causing low affinity of the channel to TTX [41]. Experimental feeding of the cultured non-toxic puffers with TTX-containing diet resulted in fish immune activation [42]. In this study, one of the previously non-toxic *L. spadiceus* species harbored TTX in liver. The mechanism that confers TTX tolerability of the previously non-toxic puffers requires investigation. 

The finding that all of the fish from the Andaman sea were toxic and all adult *T. nigroviridis* contained higher amounts of tissue accumulated TTX indicates that there may be more production and/or bio-concentration of the TTX in *T. nigroviridis* and *A. reticularis*, from the Andaman seas, than either species from the Gulf of Siam. The two *A. reticularis* were young fish thus the amounts of accumulated TTX were not as high as in the *T. nigroviridis.* The poorly developed reproductive tissue of the juvenile *A. reticularis* did not contain detectable TTX which conforms to the notion that TTX tend to accumulate in the reproductive tissue of mature fish (ovaries/eggs), especially in spawning season [33]. It is also noteworthy that the *Arothron* spp. is a common toxic puffer among the intoxicated people of the Philippines [43] which shares the Andaman seas with Thailand.

A decade ago (1992-1993) nine species of marine puffers were caught from the Andaman seas including *Arothron immaculates*, *A. stellatus*, *Chelonodon patoca*, *Diodon hystrix*, *Lagocephalus lunaris*, *L. inermis*, *L. spadiceus*, *L. sceleratus*, and *Xenopterus naritus*. They were landed at the fish ports in Phuket, Trang, and Ranong provinces located at the western coast in Southern Thailand. After testing the muscle, liver, skin and eggs of individual fish by a standard mouse bioassay [35], it was found that five species contained the TTX. They were: *A. immaculates*, *C. patoca*, *L. lunaris*, *L. sceleratus* and *X. naritus.* The other four species, *A. stellatus*, *D. hystrix*, *L. inermis* and *L. spadiceus,* did not contain detectable TTX [24,32]. In the present study, neither of the previously reported nine puffer species could be caught from the Andaman seas although several fishing by several pair trawls and push nets had been attempted by the co-operated fishermen of this study. The Andaman seas experienced a disastrous Tsunami in 2004 from the earthquake in the Indian Ocean (~9.3 Richter) which possibly affected the ecology of the marine organisms as well as puffer species. The toxic *Tetraodon* spp. reported previously in Thailand were *Tetraodon leiurus*, *T. fangi* (*T. cochinchinensis*), *T. palembangenesis* which were from brackish or fresh water [30,31]. The finding in this study that all of the marine *T. nigroviridis* from the Andaman seas were highly toxic is the first report of the toxin from a marine species of *Tetraodon*. In addition, *A. reticularis* fish is another new puffer species to be caught from the Thai seas. 

LC results revealed that commercial standard TTX and the puffer tissue extracts also contained TTX which gave rise to, more or less, the same fragmentation products after MS/MS. The retention time of standard TTX was not identical to those of puffer fish TTX. The difference was probably due to less purity of the puffer TTX which contained also other tissue substances. Impurity affects Tm (Tm of fish samples was shorter than Tm of standard TTX) and also retention time. The standard TTX and the liver of *T. nigroviridis* contained similarly anhydro and deoxyTTX. From the MS/MS result of standard TTX as well as puffer extracts, an increasing of size of fragmented product was found. This can occur by adduct ions. Common adduct ions in positive ionization mode are Na^+^, NH_4_^+^, and K^+^ [44,45,46]. The observed adduct ions in the result may be Na^+^ adduction of *m*/*z* 320 ([M + Na]^+^ = 341.9) or K^+^ adduction of *m*/*z* 302 ([M + K]^+^ = 340.9). The evidence of adduct sodium ion of tetrodotoxin was previously reported by Quilliam *et al.* in 1989 [47]. 

Our findings not only demonstrated the variation in the distribution of TTX-bearing puffers in the Andaman sea, but also raised a concern that people consuming puffers, and perhaps other seafoods, not only Thais but also inhabitants of Bengal, India, Myanmar, and Malaya, are at an extremely high risk to the deadly TTX intoxication. 
